# N-Cadherin Dependent Collective Cell Invasion of Prostate Cancer Cells Is Regulated by the N-Terminus of α-Catenin

**DOI:** 10.1371/journal.pone.0055069

**Published:** 2013-01-24

**Authors:** Yuanyuan Cui, Soichiro Yamada

**Affiliations:** Department of Biomedical Engineering, University of California Davis, Davis, California, United States of America; China Medical University, Taiwan

## Abstract

Cancer cell invasion is the critical first step of metastasis, yet, little is known about how cancer cells invade and initiate metastasis in a complex extracellular matrix. Using a cell line from bone metastasis of prostate cancer (PC3), we analyzed how prostate cancer cells migrate in a physiologically relevant 3D Matrigel. We found that PC3 cells migrated more efficiently as multi-cellular clusters than isolated single cells, suggesting that the presence of cell-cell adhesion improves 3D cell migration. Perturbation of N-cadherin function by transfection of either the N-cadherin cytoplasmic domain or shRNA specific to N-cadherin abolished collective cell migration. Interestingly, PC3 cells do not express α-catenin, an actin binding protein in the cadherin complex. When the full-length α-catenin was re-introduced, the phenotype of PC3 cells reverted back to a more epithelial phenotype with a decreased cell migration rate in 3D Matrigel. Interestingly, we found that the N-terminal half of α-catenin was sufficient to suppress invasive phenotype. Taken together, these data suggest that the formation of N-cadherin junctions promotes 3D cell migration of prostate cancer cells, and this is partly due to an aberrant regulation of the N-cadherin complex in the absence of α-catenin.

## Introduction

Cancer cell invasion is the critical first step of metastasis and the phenotypic transition from benign tumor to invasive cancer requires changes in the gene expression profile. For epithelial-derived cancers, this epithelial-to-mesenchymal transition is initiated by transcription factors that down-regulate tumor suppressors and up-regulate oncogenes, and is thought to govern cancer metastasis [1]. The key epithelial and mesenchymal markers that define the respective phenotypes are epithelial (E) and neuronal (N) cadherins, and this cadherin switch often coincides with the transition from benign to aggressive cancers [2].

In various cancer cells, the abnormal expression of N-cadherin correlates with the induction of cell motility. For example, the expression of N-cadherin induces cell migration in breast cancer cells [3]–[7], melanoma [8], prostate cancer [9], gastric cancer [10] and squamous carcinoma [11]. Interestingly, overexpression of N-cadherin enhances cell motility and invasion without decreasing E-cadherin levels [4], suggesting that increased cell motility is due to the expression of N-cadherin rather than a lack of E-cadherin. Therefore, the tight regulation of N-cadherin expression is essential in normal epithelial cell function. Consistent with this notion, the regulation of N-cadherin by microRNA-145 has been shown to suppress invasion and metastasis in gastric cancer [10].

While the canonical function of N-cadherin is to establish cell-cell adhesion, the presence of N-cadherin also induces pro-migratory signaling. The extracellular domain of N-cadherin interacts with FGF-receptor 1 [12], and this interaction minimizes the receptor internalization, thereby prolonging MAPK-ERK activation [5], [6]. Furthermore, N-cadherin-induced cell migration is dependent on reduced Akt3 level and activation in breast cancer cells [7]. In contrast, the role of N-cadherin-mediated cell-cell adhesion in cancer cell migration is unclear. If N-cadherin junctions function similarly to E-cadherin junctions by stabilizing cell-cell interactions and preventing cell migration (contact inhibition), then N-cadherin junctions would hinder cancer cell migration. Therefore, such cellular junctions would be counter-productive to N-cadherin induced pro-migratory signals.

Using prostate cancer cell lines as a model system, we sought to analyze how N-cadherin regulates cancer cell invasion. In prostate cancer, N-cadherin expression is up-regulated and E-cadherin expression is down-regulated [13], [14]. A similar cadherin switch is also associated with clinical recurrence [15], and was identified in metastatic lesions [16]. Furthermore, N-cadherin levels increased in castration-resistant tumors in patients with established metastases [17]. In addition, elevated levels of N-cadherin were observed in high grade prostate tumors compared to low grade tumors [18]. Treatment with an N-cadherin-specific monoclonal antibody reduced proliferation, adhesion and invasion of prostate cancer cells *in vitro*, and slowed the growth of multiple established xenografts, blocked local invasion and metastasis and, at higher doses, led to complete regression [9]. Taken together, these studies highlight the relationship between N-cadherin expression and prostate cancer progression, however, the specific role of N-cadherin junctions during cancer cell invasion is unknown.

Previous studies focused on cancer cell migration on a two-dimensional (2D) substrate, while little is known about how prostate cancer cells migrate in a more physiologically relevant three-dimensional (3D) matrix. Using time-lapse microscopy, we found that in 3D Matrigel, prostate cancer (PC3) cells migrate in multicellular clusters, and this collective cell migration of PC3 cells is enhanced in the presence of cell-cell adhesion. Prostate cancer cells over-expressing the N-cadherin cytoplasmic domain or N-cadherin knockdown cells did not migrate in a 3D matrix. Furthermore, as PC3 cells lack endogenous α-catenin, re-expression of α-catenin promoted an epithelial phenotype in a 3D matrix. Taken together, these results suggest that N-cadherin junctions promote 3D collective cell migration of prostate cancer cells in part due to the absence of α-catenin.

## Results

### Collective cell migration is a unique property of PC3 cells in 3D matrix

**Figure 1:**
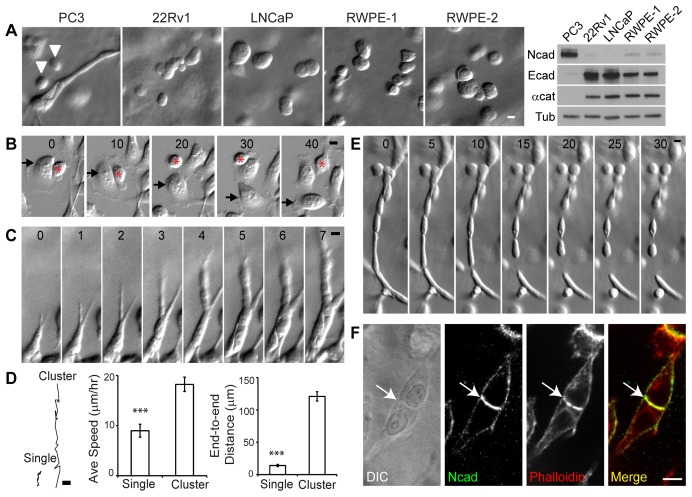
Cell-cell interaction improves the efficiency of PC3 cell migration in 3D Matrigel. (A) Morphology of prostate cancer cell lines in 3D Matrigel. PC3 cells form multi-cellular invasive cluster but 22Rv1, LNCaP, RWPE-1 and RWPE-2 cells form multi-cellular spheroids and are not invasive. Arrowheads indicate single, non-migratory PC3 cells. Right panel shows immunoblots for N-cadherin (Ncad), E-cadherin (Ecad), α-catenin (α-cat) and tubulin (Tub) of prostate cancer cell lines. (B) Cell migration on 2D surface. Red star and black arrow separately mark and track two neighboring cells passing each other without forming and maintaining cell-cell contact. Time in minutes. (C) Time-lapse images of collective cell migration of PC3 cells in 3D Matrigel. Time in hours. (D) Representative trajectories (left), average speed (middle) and end-to-end displacement (right) of single cells (N = 33) and multicellular clusters (N = 43) in 3D Matrigel (**, p<0.01). (E) EDTA addition disrupts cell-cell interactions. EDTA was added at a final concentration of 1.4 mM. Time in minutes. (F) Immunostaining for N-cadherin in PC3 cells seeded in 3D Matrigel. N-cadherin (clone 6G11) co-localizes with actin (phalloidin) at cell-cell contacts. Arrow indicates cell-cell contact. All scale bars are 10 µm.

Unlike the rigid 2D coverslips often used to study cancer cell migration, 3D Matrigel provides a soft matrix that better mimics the tumor microenvironment. We compared the migratory phenotype of prostate cancer cell lines (PC3, 22Rv1, LNCaP, RWPE-1 and RWPE-2) in 3D matrix. Only PC3 cells invaded the surrounding matrix efficiently, while 22Rv1, LNCaP, RWPE-1 and RWPE-2 cells remained round and formed spherical cell clusters with no obvious membrane extensions (Figure 1A). Interestingly, in 3D matrix, PC3 cells migrated only when in contact with neighboring cells (Figure 1A, white arrowheads point to single non-migratory cells). In addition, PC3 cells were highly migratory on the surface of Matrigel-coated coverslips (Figure 1B, Movie S1), however, the cells did not maintain cell-cell contacts on the 2D surface and migrating cells passed each other without forming stable cell-cell adhesions (Figure 1B).

In 3D Matrigel, elongated PC3 cell clusters migrated in a persistent direction (Figure 1C, D, Movie S2), while single cells migrated randomly with a lower average speed and net displacement than the individual cells within cell clusters (Figure 1D). These data suggest that collective cell migration of PC3 cells are unique to migrating cells in 3D Matrigel, and the presence of cell-cell interactions may promote efficient cell migration (cell speed and persistency) in 3D Matrigel.

### Cadherins are potential regulators of collective cell migration

Since many cell-cell adhesion proteins are calcium-dependent, we analyzed collective cell migration in 3D Matrigel under low calcium conditions. Upon chelating calcium with EDTA, PC3 cells in elongated clusters dissociated from neighboring cells over the course of 30 minutes (Figure 1E, Movie S3). This suggests that calcium-dependent adhesion proteins (e.g. cadherins) are likely responsible for PC3 cell-cell interactions. Interestingly, PC3 cells expressed higher N-cadherin and lower E-cadherin levels compared to the 22Rv1, LNCaP, RWPE-1 and RWPE-2 prostate cancer cell lines (Figure 1A). Furthermore, only PC3 cells invaded the 3D matrix whereas the other prostate cancer cell lines did not (Figure 1A). In 3D Matrigel, N-cadherin localized to cell-cell contacts of PC3 cells (Figure 1F), suggesting that N-cadherin mediates cell-cell interactions in 3D matrix.

**Figure 2:**
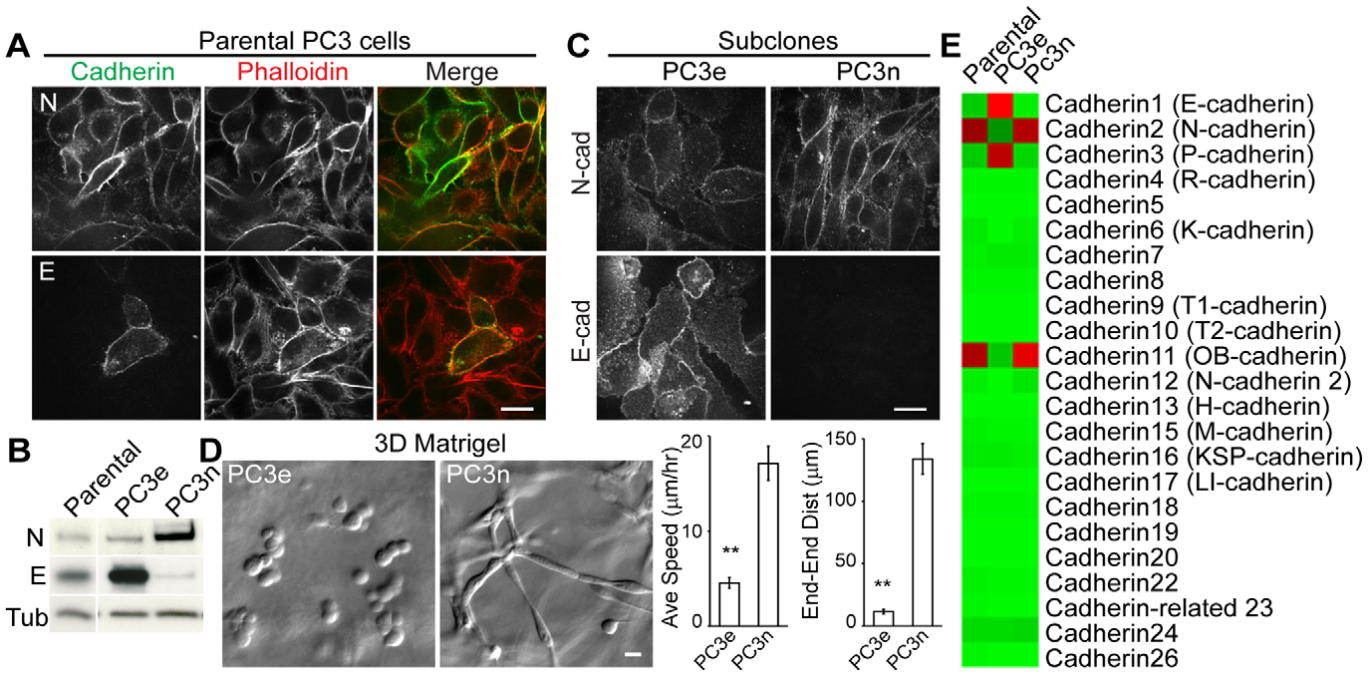
N-cadherin expression levels correspond with increased cell migration in 3D Matrigel. (A) Immunostaining of N-cadherin with phalloidin (top panel) and E-cadherin with phalloidin (bottom panel) of parental PC3 cells plated on coverslips. (B) Two representative subclones from parental PC3 cells, PC3e and PC3n, were immunoblotted for E-cadherin and N-cadherin. Tubulin is shown as a loading control. The original blots are shown in Supplementary Figure S1. (C) Immuno-staining of PC3 subclones for E-cadherin and N-cadherin (clone 32). (D) Cell morphologies, cell speed and directional persistency of PC3e (N = 19) and PC3n (N = 20) clones in 3D Matrigel (**, P<0.01; N = 11–42). All scale bars are 20 µm. (E) Heat map of microarray analysis for all cadherin expression levels in parental PC3, PC3e and PC3n cells.

The immunolabeling of N and E-cadherin revealed that most PC3 cells were N-cadherin positive (Figure 2A), but a very few PC3 cells were E-cadherin positive (Figure 2A). Interestingly, we observed that, while the majority of PC3 cells collectively migrated in 3D Matrigel, a few cells remained in spherical cell clusters. We suspected that non-migratory PC3 cells may be expressing E-cadherin. To accurately assess the role of both cadherins in 3D cell migration, PC3 cells were subcloned to isolate E-cadherin expressing PC3 cells. Of twenty-one PC3 subclones generated, most subclones had higher or comparable level of N-cadherin to the parental cells (see Figure S1). Two sub-clones were chosen (PC3e and PC3n) for their characteristic cadherin expression. PC3e had a higher E-cadherin expression level than both the parental PC3 and PC3n, while PC3n had a higher expression level of N-cadherin (Figure 2B, C, S1). E and N cadherin proteins localized to cell-cell contacts of PC3e and PC3n clones, respectively (Figure 2C). Furthermore, the cadherin expression analysis revealed that the parental and PC3n clone also expressed a high level of cadherin 11, whereas PC3e primarily expressed E and P-cadherin (Figure 2E). In 3D Matrigel, PC3e cells did not migrate and remained as spherical clusters (Figure 2D, Movie S4). In contrast, PC3n had an elongated morphology and migrated in a persistent direction similarly to the parental PC3 cells (Figure 2D, Movie S5). These data suggest that the expression levels of E/P and N/11-cadherins correspond with the spherical and elongated phenotype in 3D matrix, respectively.

### Expression of the N-cadherin cytoplasmic domain abolishes collective cell migration

**Figure 3:**
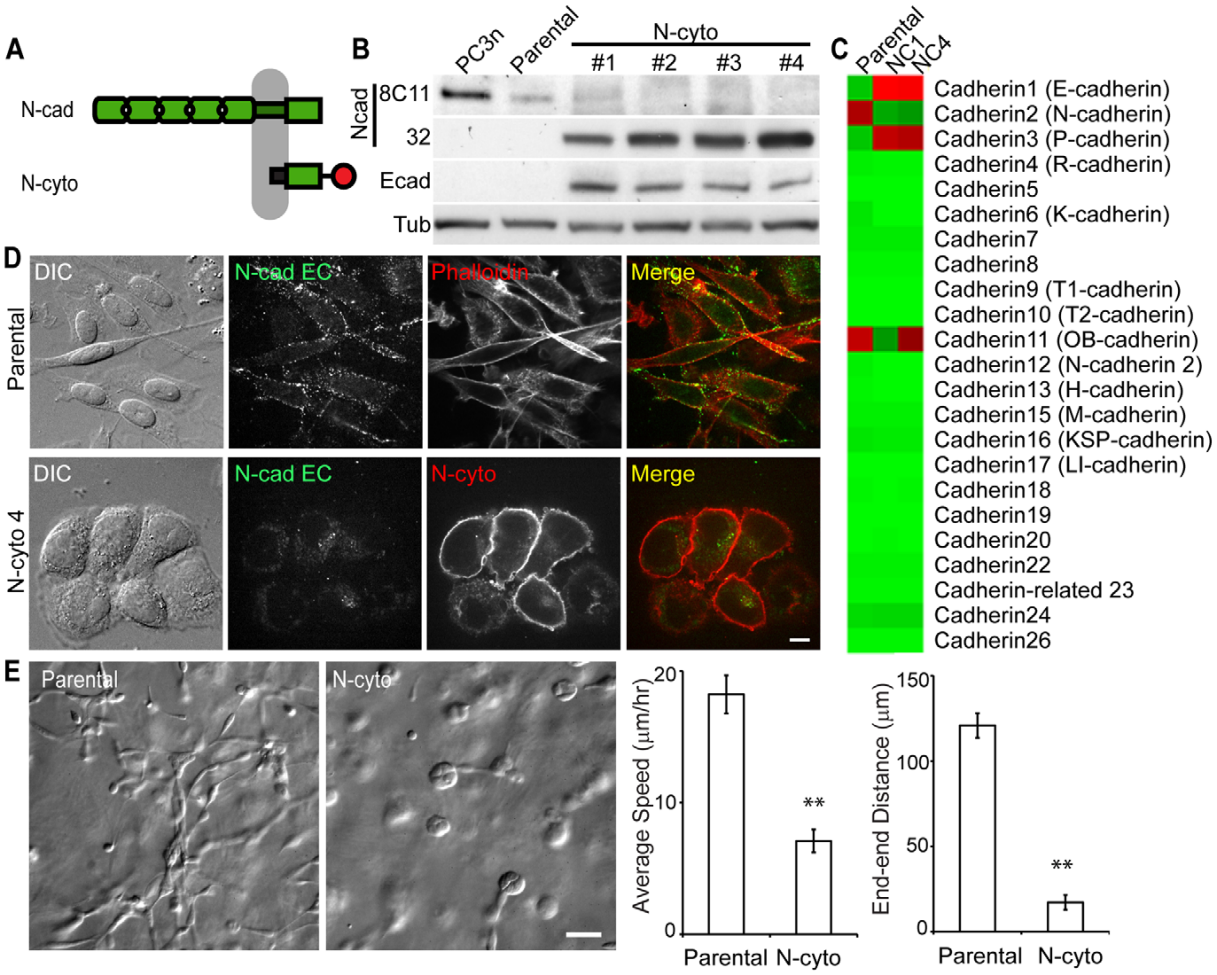
Overexpression of the cytoplasmic domain of N-cadherin reduces 3D cell migration. (A) Schematic of the full-length N-cadherin and the cytoplasmic N-cadherin (N-cyto) constructs. N-cadherin cytoplasmic domain is fused with membrane targeting domain from Lyn kinase (black) at the N-terminus and tandem dimer-tomato (red) at the C-terminus. (B) Immunoblots for N-cadherin extracellular domain (clone 8C11), N-cadherin cytoplasmic domain (clone 32), and E-cadherin (clone 36) in N-cyto expressing PC3 cell subclones. #1, #2, #3, #4 are four populations of N-cyto cells with gradually increased N-cyto levels. (C) Heat map of microarray analysis for all cadherin levels in PC3, N-cyto #1 and N-cyto #4 cells. (D) Immuno-staining of extracellular domain of N-cadherin (clone 8C11), with phalloidin in parental PC3 cells (top panel) and N-cyto #4 cells (bottom panel). N-cyto localization was detected by the tandem-tomato signal (bottom panel). Scale bar 10 µm. (E) Statistical analysis of N-cyto expressing PC3 cell 3D migration. Average speed and end to end distance are shown (**, P<0.01; N = 42 for parental cells and N = 11 for N-cyto cells). Error bars are standard error of the mean. Scale bar, 40 µm.

To analyze the role of N-cadherin in collective cell migration, we transfected N-cadherin cytoplasmic domain (N-cyto), and subcloned the stable transfectants with increasing N-cyto levels (Figure 3A). In the presence of N-cyto, endogenous N-cadherin expression was down-regulated, while endogenous E-cadherin was up-regulated (Figure 3B). Microarray analysis confirmed the decreased N-cadherin and increased E-cadherin expression profile in N-cyto cells (Figure 3C), and further revealed the up-regulation of P-cadherin (Figure 3C). Both E and P-cadherin were also up-regulated in the non-migratory PC3e clone (Figure 2E). Taken together, these data demonstrate that the down-regulation of N-cadherin results in the up-regulation of both E and P-cadherins in PC3 cells. Interestingly, the cadherin 11 was down-regulated in one of the N-cyto expressing cells (#1) but not in others (#4, Figure 3C).

Instead of a loosely organized and elongatged morphology on a 2D surface, N-cyto expressing cells had tighter cell-cell contact than parental cells. Immunostaining for the extracellular domain of N-cadherin detected low levels of endogenous N-cadherin that were not localized at cell-cell contacts (Figure 3D). In 3D Matrigel, both N-cyto #1 and #4 cells formed spherical clusters and were not invasive (Figure 3E, Movie S6). Although these two N-cyto expressing clones shared the same non-invasive phenotype in 3D Matrigel, they have distinct cadherin 11 levels, suggesting that cadherin 11 expression is independent of the PC3 cell migration phenotype and behavior in 3D Matrigel.

### Silencing N-cadherin abolishes cell migration in 3D Matrigel

**Figure 4:**
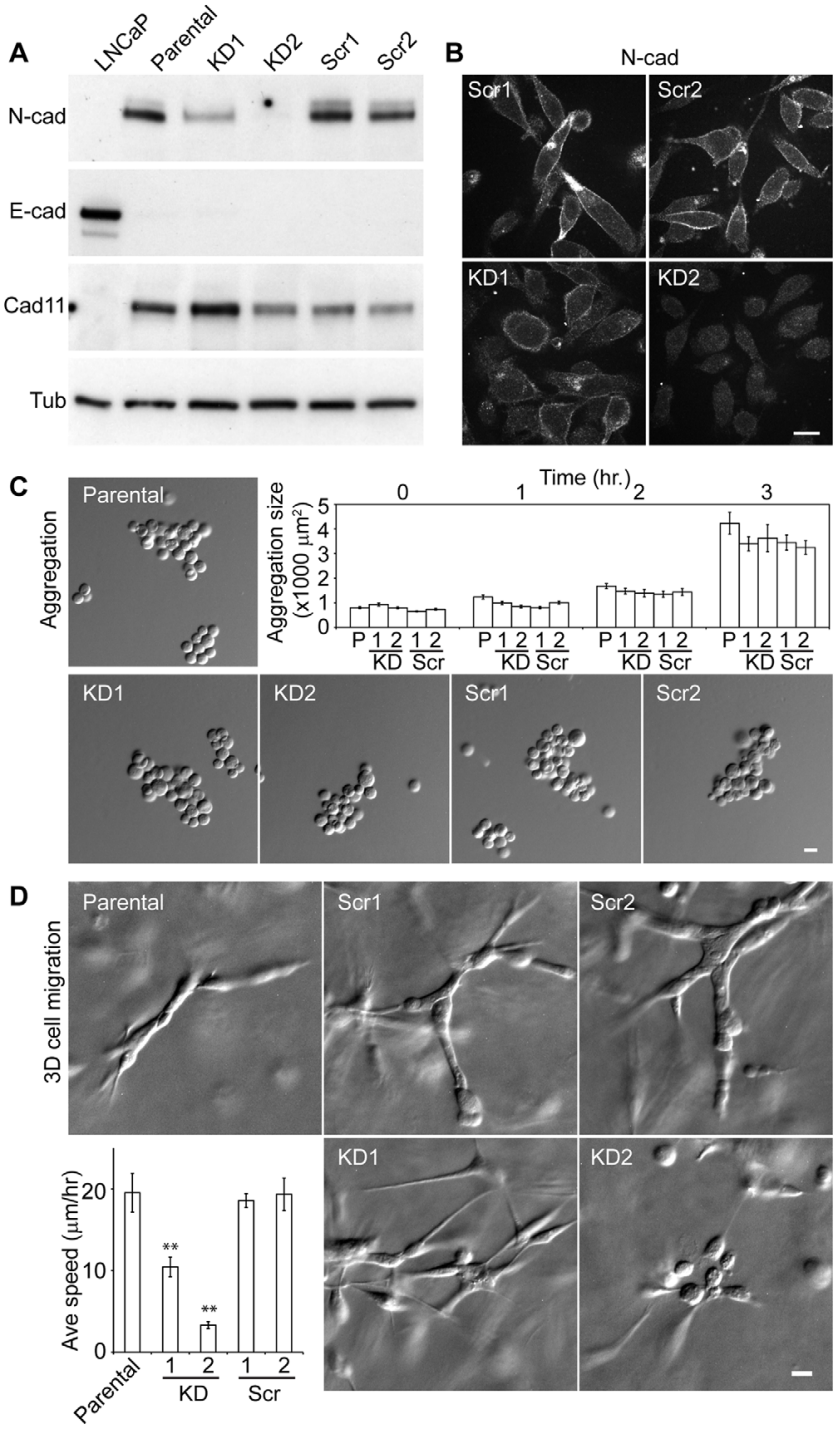
N-cadherin deficient cells are non-migratory in 3D Matrigel. (A) Immunoblots for N-cadherin, E-cadherin and cadherin 11 in N-cadherin knockdown cells and cells transfected with scrambled sequences. Tubulin is shown as a loading control. KD1, KD2 are two knockdown clones. Scr1, Scr2 are two clones transfected with corresponding scrambled sequences. (B) Immunostaining of N-cadherin in control cells (top panel) and knockdown cells (bottom panel). (C) Cell aggregate formation of knockdown cells and control cells over 3 hours in suspension. At hour 0, 1, 2, 3 in suspension, the numbers of cell aggregates analyzed were 144, 101, 133, 62 for parental PC3 cells, 147, 117, 107, 81 for KD1 cells, 142, 121, 107, 73 for KD2 cells, 146, 115, 87, 88 for Scr1 cells, 131, 147, 107, 68 for Scr2 cells, respectively. (D) 3D cell migration analyses of parental (N = 17), scramble shRNA (N = 17 for Scr1, N = 20 for Scr2) and knockdown cells (N = 37 for KD1, N = 33 for KD2). Statistical analysis of average speed is shown (**, P<0.01). All error bars are standard error of the mean. All scale bars are 20 μm.

To further determine whether N-cadherin is the primary regulator of 3D cell migration, we stably silenced N-cadherin in PC3 cells. Of three N-cadherin specific shRNA sequences tested and 44 transfected clones analyzed, two clones with 50% (KD1) and 99% (KD2) reduction of endogenous N-cadherin are shown. The control group with corresponding scrambled sequences (Scr1, Scr2) showed similar N-cadherin levels to parental PC3 cells (Figure 4A, B). Residual endogenous N-cadherin was observed in KD1 cells, but not in KD2 cells (Figure 4B). Moreover, silencing of N-cadherin did not change E-cadherin and cadherin 11 levels in KD1 and KD2 cells (Figure 4A).

Despite the decreased level of N-cadherin, the knockdown cells still formed cell aggregates similar in size as parental PC3 cells in suspension (Figure 4C), suggesting that the presence of other cadherins, e.g. cadherin 11, may be sufficient for cell-cell contact formation. However, in 3D Matrigel, the partially knockdown (KD1) cells elongated similarly to the parental cells, but formed much looser cell clusters (Figure 4D). The complete knockdown (KD2) cells remained round with minimal membrane extensions in 3D Matrigel (Figure 4D). Unlike the parental or scrambled shRNA transfected cells, these cells did not develop large elongated cell clusters (Figure 4D).

In 3D Matrigel, the both knockdown cell lines did not migrate as fast as the parental or scrambled shRNA transfected cells. While KD1 cells migrated with a 50% reduction in speed, KD2 cells were almost immobile (Figure 4D, Movie S7). These data suggest that, in 3D Matrigel, N-cadherin regulates both the elongated morphology and development of cell clusters, in addition to cell migration.

### Expression of α-catenin reverts PC3 cells to normal epithelial phenotype

**Figure 5:**
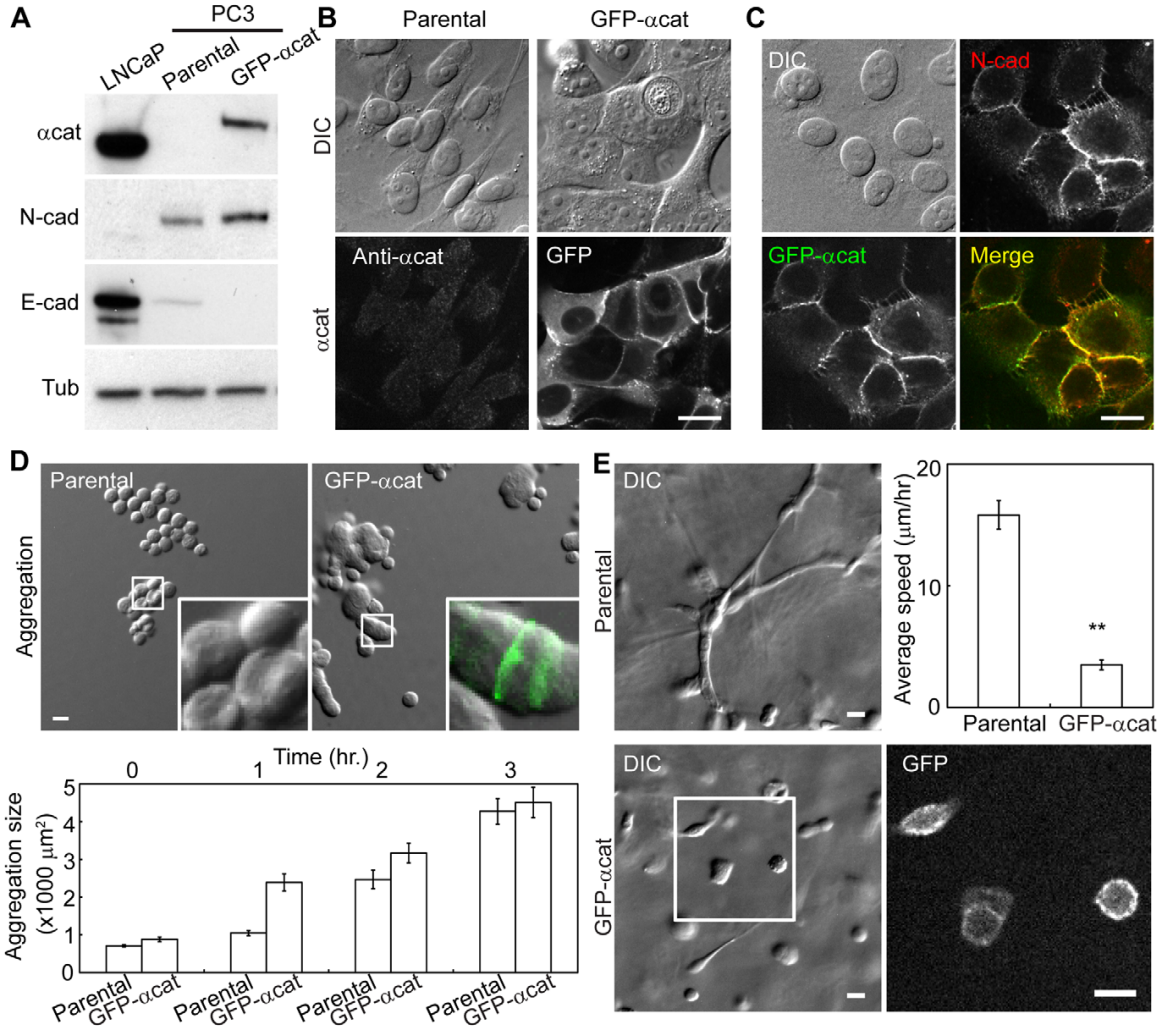
Expression of α-catenin strengthens cell-cell adhesion and decreases 3D cell migration. (A) Immunoblots for α-catenin, N-cadherin and E-cadherin in α-catenin expressing cells. Tubulin is shown as a loading control. LNCaP was used as a positive control for α-catenin and E-cadherin. (B) Localization of α-catenin in parental PC3 cells and GFP-tagged α-catenin expressing cells in bottom panels. (C) Co-localization of GFP-tagged α-catenin with N-cadherin. (D) Cell aggregation analysis of parental and α-catenin expressing cells in suspension. Inset: enlarged views of cell clusters. GFP signal is overlayed onto bright-field image for GFP-tagged α -catenin expressing cells. Average aggregation sizes at each time points are shown in the bar graph. (E) Cell migration analyses in 3D Matrigel. GFP channel of GFP-tagged α-catenin expressing cells in the white box (bottom left panel) is shown in bottom right panel. Statistical analysis of cell migration speed is shown (**, P<0.01; N = 35 for parental cells, N = 31 for GFP-α-catenin expressing cells). All scale bars are 20 μm.

Cadherins play a critical role in forming and maintaining strong cell-cell adhesions and require support from the underlying actin cytoskeleton to maintain cell-cell contacts. One key member of the cadherin complex, α-catenin, contains an actin binding domain at the C-terminus. Interestingly, unlike 22Rv1 or LNCaP cells, PC3 cells do not express α-catenin (Figure 1A, 5A). To understand how α-catenin expression affects N-cadherin dependent collective cell migration, we stably transfected GFP-tagged α-catenin into PC3 cells (Figure 5A, B). The GFP- α-catenin expressing cells had a slight increase in N-cadherin level and a slight decrease in E-cadherin level compared to the parental cells (Figure 5A). Interestingly, the α-catenin expressing cells formed tighter cell-cell contacts in more compact cell clusters compared to the loosely organized, elongated parental cells on 2D surface (Figure 5B). Additionally, the GFP tagged α-catenin localized to cell-cell contacts (Figure 5B), and co-localized with N-cadherin (Figure 5C).

In suspension, α-catenin expressing cell aggregates were comparable in size to parental PC3 cells (Figure 5D). However, the cell-cell contacts of α-catenin expressing cells were much tighter without obvious cell-cell boundaries than the looser parental PC3 cell aggregates (Figure 5D). The compaction of cell aggregates in α-catenin expressing cells suggests that N-cadherin mediated cell-cell adhesion is likely strengthened by the presence of α-catenin.

Despite the ability to form cell-cell adhesion in suspension (Figure 5D), α-catenin expressing PC3 cells did not elongate to form large cell clusters in 3D Matrigel as parental PC3 cells did. These cells remained as single cells or small clusters with a more round morphology (Figure 5E). In 3D Matrigel, α-catenin localized to cell-cell contacts of small cell clusters (Figure 5E). Consistent with this morphology, α-catenin cells were not motile in 3D Matrigel (Figure 5E, Movie S8). These results suggest that α-catenin expression strengthened N-cadherin mediated cell-cell adhesion and prevented cell migration in 3D Matrigel, and that down-regulation of α-catenin may be an important step in prostate cancer metastasis.

### The N-terminal half of α-catenin is sufficient for suppressing invasive phenotype

**Figure 6:**
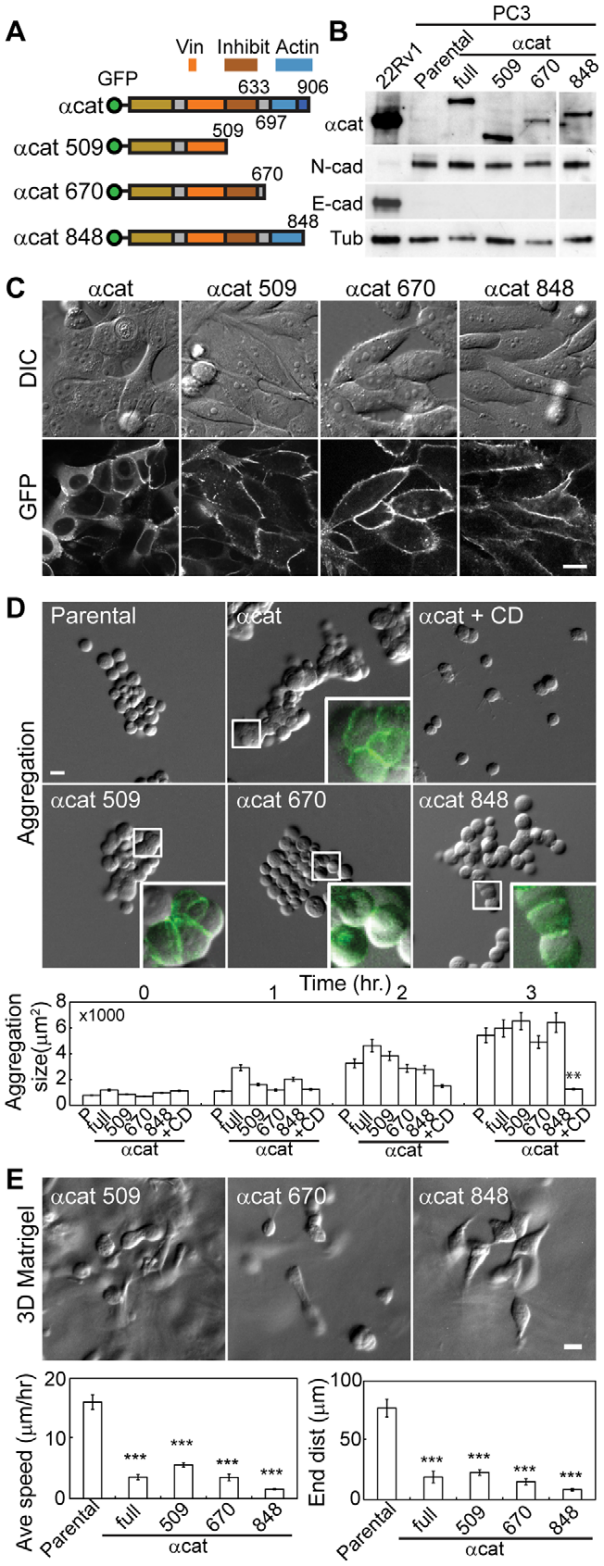
The N-terminal half of α-catenin is sufficient to suppress invasive phenotype. (A) Schematic of full-length α-catenin and its C-terminal truncated mutants. GFP tag, Vinculin binding (Vin), its inhibitory (Inhibit), F-actin binding (Actin) domains are shown. (B) Immunoblots for α-catenin, N-cadherin and E-cadherin in various α-catenin expressing cells. Tubulin is shown as a loading control. 22Rv1 was used as a positive control for α-catenin and E-cadherin. The original blots are shown in supplementary Figure S2. (C) Cell morphology (top panel) and localization of α-catenin (bottom panel) in various α-catenin expressing PC3 cells. (D) Cell aggregation analysis of parental, various α-catenin expressing cells, and 10 μM cytochalasin D (CD) treated cells. Inset: enlarged views of cell clusters. GFP signal is overlayed onto bright-field image for GFP-tagged α–catenin/truncation mutant expressing cells. Aggregation size of the cells at each time points are shown in the mean ± standard error of the mean; ANOVA analysis combined with posthoc test Tukey HSD were used between groups at timepoint hour 3 (**, P<0.01). (E) Cell migration analyses in 3D Matrigel for parental (N = 35), the full-length α-catenin (N = 31), α-catenin 1–509 (N = 73), α-catenin 1–670 (N = 39), and α-catenin 1-848 (N = 52) expressing cells. Average speed and endpoint distance of migration are shown in mean ± standard error of the mean; ANOVA and posthoc test Tukey HSD were used (***, P<0.001). All scale bars are 20 μm.

To test the roles of α-catenin in regulating cell-cell adhesion, we progressively truncated α-catenin construct (Figure 6A) and stably transfected PC3 cells. The C-terminus of α-catenin consists of vinculin binding inhibitory domain (AA 509–633), and actin binding domain (AA 697–906), which requires the short C-terminal end (AA 848–906) for actin binding [19]. The full-length α-catenin and the truncated mutants were comparable in their expression levels (Figure 6B, S2), and the expression of the truncated mutants did not change the cadherin expression profile (Figure 6B). While all truncated mutants localized to the sites of cell-cell adhesion (Figure 6C), the truncated mutant expressing cells formed looser cell clusters than that of full length α-catenin expressing cells on a 2D surface (Figure 6C). Interestingly, the truncation mutants α-catenin 1–509 and α-catenin 1-848 expressing cells formed relatively compact clusters than α-catenin 1–670 expressing cells on a 2D surface (Figure 6C).

The morphological phenotypes on a 2D surface were consistent with the results of cell aggregation analysis in suspension. While truncated mutant expressing cells formed cell aggregates comparable in size with that of parental and full-length α-catenin expressing cells, the cell aggregates showed different phenotypes (Figure 6D). Similar to the full-length α-catenin expressing cells, α-catenin 1–509 expressing cells formed tight cell-cell contact without obvious cell boundaries (Figure 6D), suggesting that the N-terminus of α-catenin is essential for this tight cell-cell adhesion. This is consistent with the α-catenin mutant that lack the C-terminal end essential for actin binding (1-848) were also capable of inducing tight cell clusters, albeit looser clusters than the full-length α-catenin expressing cells (Figure 6D). Note that the actin organization is still essential to the formation of cell-cell adhesion as the cytochalasin treatment eliminated cell aggregation formation (Figure 6D). Surprisingly, however, α-catenin 1–670 expressing cells formed loose cell aggregates similar to that of parental cells (Figure 6D).

Since the α-catenin mutant that lack the C-terminal end essential for actin binding (1–848) forms relatively tight cell clusters (Figure 6D), the direct actin binding by α-catenin has only a minor role in the regulation of cell-cell adhesion. In addition, our data suggest that, in the absence of actin-binding domain (AA 697–906), the vinculin-binding inhibitory domain suppresses the ability to form tight cell-cell junctions, which is consistent with the notion that vinculin is required for strengthening of cell-cell adhesion [20], [21]. In support of this model, minimal vinculin recruitment was observed along the cell-cell contacts of α-catenin 1–670 expressing cells (Figure S3).

Despite different phenotypes in suspension, all truncated mutant expressing cells obtained similar epithelial morphology to that of full length α-catenin expressing cells in 3D Matrigel (Figure 6E). The α-catenin mutant-expressing cells remained as single cells or formed small clusters with minimum membrane extensions, and were not motile in 3D Matrigel (Figure 6E). While these α-catenin mutant-expressing cells have different levels of cell-cell adhesions (Figure 6D), the expression of N-terminal half of α-catenin was sufficient to minimize cell invasion in 3D Matrigel, suggesting that cell invasiveness is determined by the protein interactions at the N-terminal half of α-catenin (e.g. ß-catenin).

## Discussion

Previous studies demonstrated that N-cadherin induces pro-migratory signaling by directly interacting with FGF-receptors at the extracellular domain [5], activates the MAPK/ERK signaling pathway [5], [6], and suppresses Akt3 signaling [7]. Yet, the role of N-cadherin-mediated cell-cell adhesion in prostate cancer cell invasion has not been explored. Our data suggest that the presence of N-cadherin-mediated cell-cell adhesion promotes efficient cell migration by increasing cell speed in a persistent migratory path in 3D Matrigel.

This collective cell migration of PC3 cells is a unique migration phenotype only shown in a 3D matrix. While N-cadherin localized to cell-cell contact of PC3 cells plated on a 2D surface (Figure 2A, 3D and 4B), these cells do not maintain stable cell-cell contacts and migrate as single cells (Figure 1B). Since the expression of key signaling molecules are different in 2D vs 3D environment [22], such factors may alter cell-cell adhesion and migration phenotype. Alternatively, the difference in 2D vs 3D cell-cell adhesion may be the results of the matrix elasticity. For example, unlike in 3D matrix, two contacting cells moving in an opposite direction can often mechanically disrupt cell-cell adhesions on a 2D surface. This is because the stiff 2D substrate provides a better traction than soft 3D matrix. The distinct migration phenotype in 3D matrix suggests that N-cadherin junctions may play an important role in prostate cancer cell invasion *in vivo*.

Unlike E-cadherin-mediated cell-cell adhesions that prevent cell migration (contact inhibition, see 22Rv1, LNCaP and RWPE in Figure 1A), N-cadherin-mediated cell-cell adhesions promote collective cell migration. One unique feature of N-cadherin adhesion is to suppress random membrane protrusions of follower cells so that only the leader cells can extend the leading edges. This local contact inhibition maintains cell polarity with a distinct leading edge for cell migration; therefore, ensuring the persistency of collective cell migration. Cadherin-mediated cell-cell adhesion has been shown to generate polarized cell morphology in in Xenopus mesendoderm cells [23] and transformed epithelial cells in a 3D matrix [24]. N-cadherin junctions in epithelial derived cancer cells provide a polarization cue essential for directional cell migration in a 3D matrix.

Interestingly, cadherin 11, a type II cadherin, is also up-regulated in the highly invasive clone of PC3 cells (PC3n, Figure 2E), and may be responsible for the formation of cell-cell adhesions in N-cadherin deficient cells (Figure 4C). This cadherin 11 up-regulation is thought to promote cellular interactions between cancer cells and cadherin 11 expressing osteoblasts that is typical of bone metastasis [25]–[27]. Previous studies have reported that the expression of cadherin 11 shRNA decreases cell migration of PC3 cells [26]. However, the presence of cadherin 11 alone is not sufficient for 3D cell migration. For example, N-cadherin deficient but cadherin 11 expressing cells are not migratory in a 3D matrix (see Figure 4). Therefore, the expression of both N-cadherin and cadherin 11 or the potential interactions between these two distinct cadherins may be essential for prostate cancer cell migration.

The E-to-N cadherin switch observed in PC3 cells is commonly observed in various cancers [2]. Often, cancerous cells down-regulate normally expressed cadherins (E-cadherin in the case of prostate), and up-regulate mesenchymal cadherins (e.g. N-cadherin, cadherin 11 etc). However, during embryogenesis, neuro crest cell migration is initiated by the down-regulation of N-cadherin [28], therefore, the N-cadherin level is not the sole determinant of the cell migratory phenotype in all processes. Consistent with this notion, an N to E-cadherin switch occurs in ovarian cancer [29], [30] while the expression of cadherin 11 has been shown to suppress cell migration [31]. Ectopic expression of new cadherins along with other cadherin regulators may be the key to controlling cell-cell adhesion of migratory cells.

Since cadherin regulation is in part mediated by the actin cytoskeleton, a cadherin switch may alter how cadherin complexes interact with the actin cytoskeleton. The key actin regulator in the cadherin complex is α-catenin, an actin binding protein that have been shown to bundle actin filaments [32], suppress the Arp2/3 mediated actin assembly [33], and act as a mechano-sensing module at cadherin junctions [20]. In prostate cancer, loss of α-catenin is frequently observed and thought to have a prognostic value [34]–[38].

Due to homozygous deletion of the gene, α-catenin is absent in PC3 cells (see Figure 5A) [39]. Introduction of GFP-tagged α-catenin restores the epithelial phenotype in PC3 cells and decreases 3D cell migration (Figure 5). This is consistent with the data that the cytoplasmic deletion of N-cadherin also weakens cell-cell adhesion in 3D matrix [24]. As shown previously, PC3 cells adhere weakly to each other even in the absence of α-catenin (see Figure 5A) [39], however, this weak cell-cell adhesion should not be neglected as it is essential for collective cell movement in 3D Matrigel (Figure 1). While previous studies have suggested that loss of cell-cell adhesion in cancer cells aids delamination of cancer cells from normal cells, our study demonstrates that residual, albeit weak, cell-cell adhesion is a critical parameter for efficient prostate cancer cell invasion.

## Materials and Methods

### Cell lines, constructs and reagents

PC3, LNCaP and 22Rv1cells (all from ATCC) were cultured in RPMI-1640 (Invitrogen, Carlsbad, CA, USA) medium supplemented with 10% fetal bovine serum, penicillin and streptomycin. RWPE-1 and RWPE-2 cells (from ATCC) were cultured in Keratinocyte Serum-Free Medium (Gibco), supplemented with 50 mg/ml bovin pituitary extract and 5 µg/ml EGF. The cytoplasmic domain of N-cadherin (Ncyto, AA 747–906) was membrane targeted with the myristoylation and plamitoylation sites of Lyn kinase (MGCIKSKRK) and fluorescently tagged with tandem dimer Tomato [24]. Mouse GFP-α-catenin and the truncation mutant GFP-α-catenin 670 were previously described [40]. Other truncation mutants were generated by PCR (α-catenin 1–509) or the addition of stop codon by mutagenesis (α-catenin 1–848). All constructs were sequence verified, and transfected into PC3 cells using Lipofectamine 2000 (Invitrogen) and selected with G418 (Invitrogen). The N-cadherin cytoplasmic domain expressing cells were obtained by FAC sorting (UC Davis Cancer Center Shared Resources). GFP-tagged α-catenin (12 clones), truncation mutants 1–509 (18 clones), 670 (16 clones) and 848 (37 clones) expressing cells were obtained by sub-cloning.

Antibodies used were N-cadherin cytoplasmic domain (clone 32, BD biosciences, San Jose, CA, USA and clone 6G11, Dako, Carpinteria, CA, USA), N-cadherin extracellular domain (clone 8C11, Biolegend, San Diego, CA, USA), E-cadherin (clone 36, BD biosciences), α-catenin (clone 15D9, Alexis Biochemicals, Farmingdale, NY, USA) and vinculin (clone hVIN-1, Sigma-Aldrich, St. Louis, MO, USA). AlexaFluor 568-phalloidin was used to detect filamentous actin (Invitrogen). Pharmacological inhibitor used was 10 μM cytochalasin D (EMD Chemicals, Darmstadt, Germany).

### N-cadherin knockdown constructs and stable cell lines

Human N-cadherin shRNA targeting the sequences: #1: AGTGTTCCCAAGACAATTC, #2: TCCAGTGACTATTAAGAGAAAT, #3: CTCCCTGTTAGTGTTTGACTAT, and three corresponding scrambled sequences: #1 S: GCCCATCTATAAAGGTCTA, #2 S: GAGCTAATGAACGATAAACTTT, #3 S: GTGCTCCTTCTCATTGTAGATT, were inserted into PiggyBac shRNA vector (System Biosciences, Mountain View, CA, USA) and transfected into PC3 cells with Lipofectamine 2000. We subcloned stable cell lines of N-cadherin (44 clones) and scrambled (31 clones) shRNA expressing cells using puromycin selection (0.5 µg/ml). KD1, KD2, Scr1and Scr2 cells were generated from sequence #3, #1, #3 S and #1 S, respectively.

### Microscopy and live cell imaging

Live cells were imaged on glass bottom dishes (MatTek, Ashland, MA, USA) in a temperature-controlled chamber at 37°C using a Zeiss AxioObserver equipped with a Yokogawa spinning disk confocal system, 10x and 40x objectives, 488 and 561 nm solid-state lasers, and a Cool SNAP HQ camera. The microscope system was controlled by Slidebook software (Intelligent Imaging Innovations, Denver, CO, USA).

### Immunofluorescence and analysis

Cells were seeded on Matrigel-coated cover glasses (2D) or in Matrigel (3D) (see methods in 3D cell migration assay) one or two days before fixing. Cells were fixed in 3% para-formaldehyde with 0.3% TritonX-100 for 10 min and blocked with 1% BSA containing 0.3% TritonX-100. Primary antibody dilutions were added and incubated for one hour at room temperature, and detected with AlexaFluor 488 or 568-conjugated secondary antibodies (Invitrogen).

### 3D cell migration assay

Growth factor reduced Matrigel (BD biosciences) was prepared per manufacture's protocol. Cells were dissociated by 0.05% trypsin-EDTA (Invitrogen) and re-suspended at a cell density of 2×10^6^ cells/ml. 15 µl of cell suspension was mixed with equal volume of Matrigel on ice and quickly plated onto a Matrigel-coated glass bottom dish. After 30-minute incubation in 37°C, RPMI media with feeder PC3 cells were added to the 3D gel-containing dish. Cell migration was observed after two to three days of culture in Matrigel. Migrating cells were imaged every 10 minutes for 12 hours. Using Slidebook and Excel software, the average migration rate and end-to-end distance of cell trajectory was calculated based on 10 fields of view for each cell type.

### Microarray expression analysis

Total RNA was isolated from PC3, PC3e, PC3n, N-cyto 1, and N-cyto 4 cells separately using RNeasy kit (Qiagen, Valencia, CA, USA) followed by QIAshredder (Qiagen). Sample labeling and hybridization to Affymetrix human gene 1.0 ST arrays were carried out under standard conditions at the UC Davis School of Medicine Microarray Core Facility. The raw data were processed and exported using dchip software (Dana-Farber Cancer Institute, Cheng Li Lab) to obtain detection calls and signal values. Heat map of cadherin expression levels was created using the HeatMap Builder software with dataset-normalized sorting (Stanford Microarray Database).

### Cell aggregation analysis and immunostaining

For cell clustering analysis, cultured cells were dissociated with trypsin (Invitrogen) in presence of 1.8 mM calcium to preserve cell surface adhesion proteins. Approximately 6250 cells contained in a total volume of 25 µl were suspended upside-down in the culture dish. Every hour, the cell solution was triturated 5 times before imaging. The average aggregate size was determined by object thresholding using ImageJ.

## Supporting Information

Figure S1 **Original immunoblot for** **Figure 2B** **.** The membranes were blotted with antibodies against N-cadherin, E-cadherin, or tubulin.
(TIF)

Figure S2 **Original immunoblot for** **Figure 6B** **.** The membranes were blotted with antibodies against α-catenin, N-cadherin, E-cadherin, or tubulin.
(TIF)

Figure S3 **Immuno-fluorescence labeling of vinculin for cell aggregation analysis shown in** **Figure 6D** **.**
(TIF)

Movie S1 **Cell migration of PC3 cells on a 2D surface.** Time is shown in hour:min. Scale bar is 10 μm.
(MOV)

Movie S2 **Collective cell migration of PC3 cells in a 3D Matrigel.** Time is shown in hour:min. Scale bar is 10 μm.
(MOV)

Movie S3 **Disruption of cell-cell adhesion of PC3 cells in a 3D Matrigel by chelating calcium with EDTA.** Time is shown in hour:min. Scale bar is 10 μm.
(MOV)

Movie S4 **Cell migration of PC3e clone in a 3D Matrigel.** Time is shown in hour:min. Scale bar is 20 μm.
(MOV)

Movie S5 **Cell migration of PC3n clone in a 3D Matrigel.** Time is shown in hour:min. Scale bar is 20 μm.
(MOV)

Movie S6 **Cell migration of the PC3 cells expressing the N-cadherin cytoplasmic domain in a 3D Matrigel.** Time is shown in hour:min. Scale bar is 20 μm.
(MOV)

Movie S7 **Cell migration of N-cadherin KD2 cells in a 3D Matrigel.** Time is shown in hour:min. Scale bar is 20 μm.
(MOV)

Movie S8 **Cell migration of α-catenin over-expressing PC3 cells in a 3D Matrigel.** Time is shown in hour:min. Scale bar is 20 μm.
(MOV)
