# Supplementary figures and images for: N-Cadherin Dependent Collective Cell Invasion of Prostate Cancer Cells Is Regulated by the N-Terminus of α-Catenin
Source: PLoS One. 2013 Jan 24;8(1):e55069. doi: 10.1371/journal.pone.0055069 (PMC3554680; doi:10.1371/journal.pone.0055069)

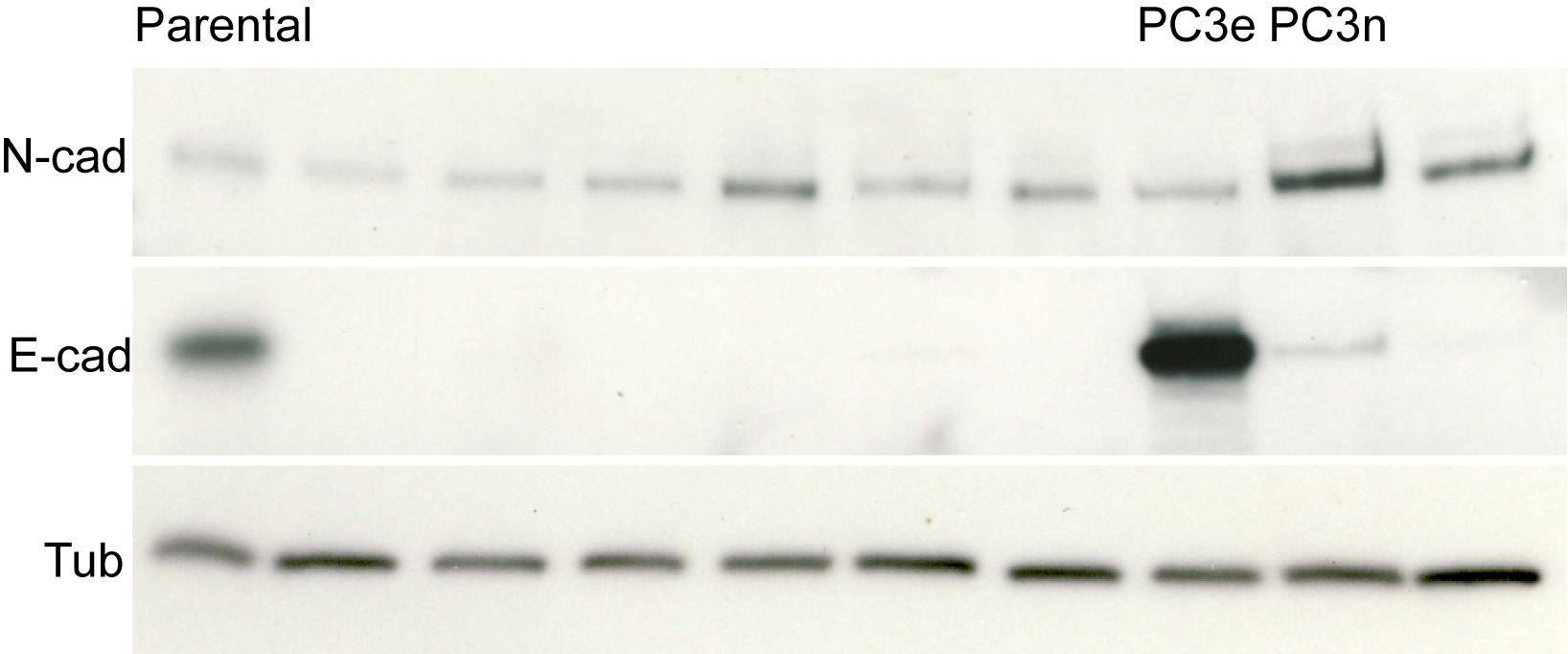

Supplement: Figure S1 — Original immunoblot for Figure 2B . The membranes were blotted with antibodies against N-cadherin, E-cadherin, or tubulin. (TIF) [file pone.0055069.s001.tif]

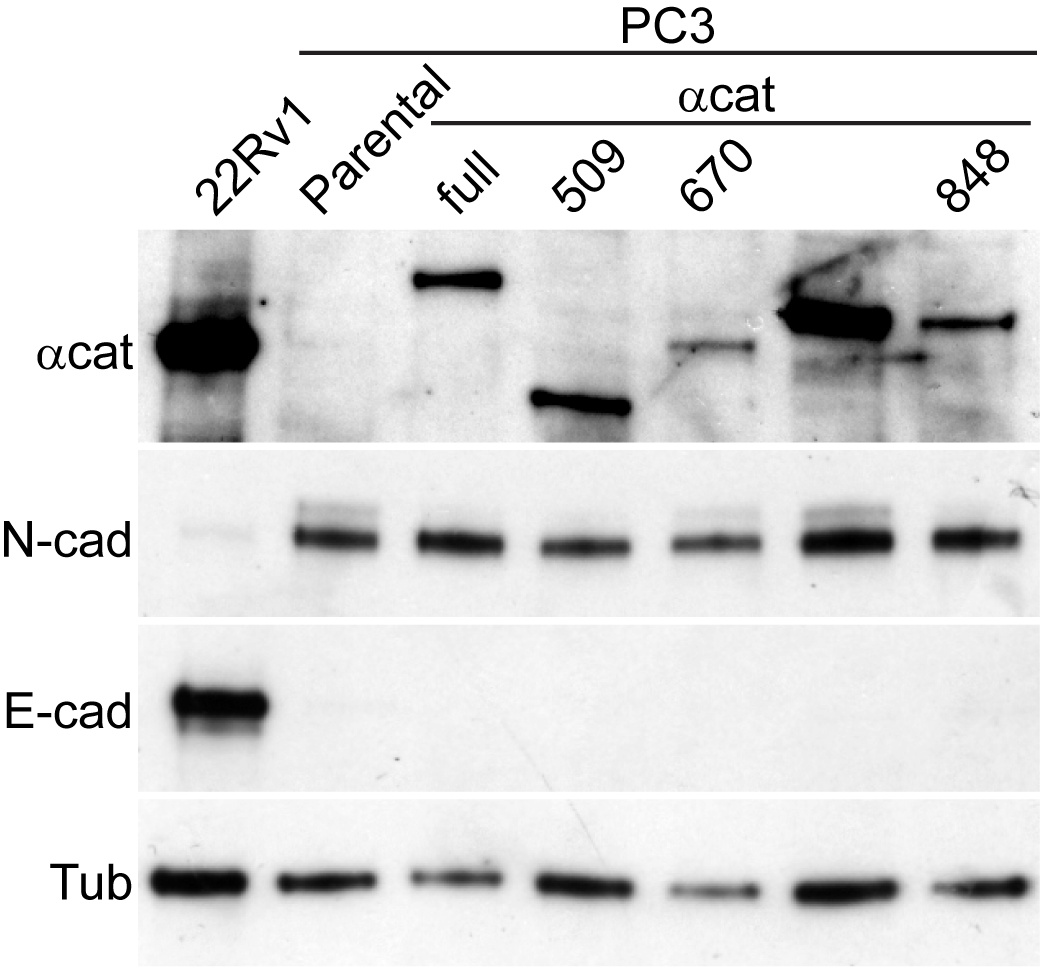

Supplement: Figure S2 — Original immunoblot for Figure 6B . The membranes were blotted with antibodies against α-catenin, N-cadherin, E-cadherin, or tubulin. (TIF) [file pone.0055069.s002.tif]

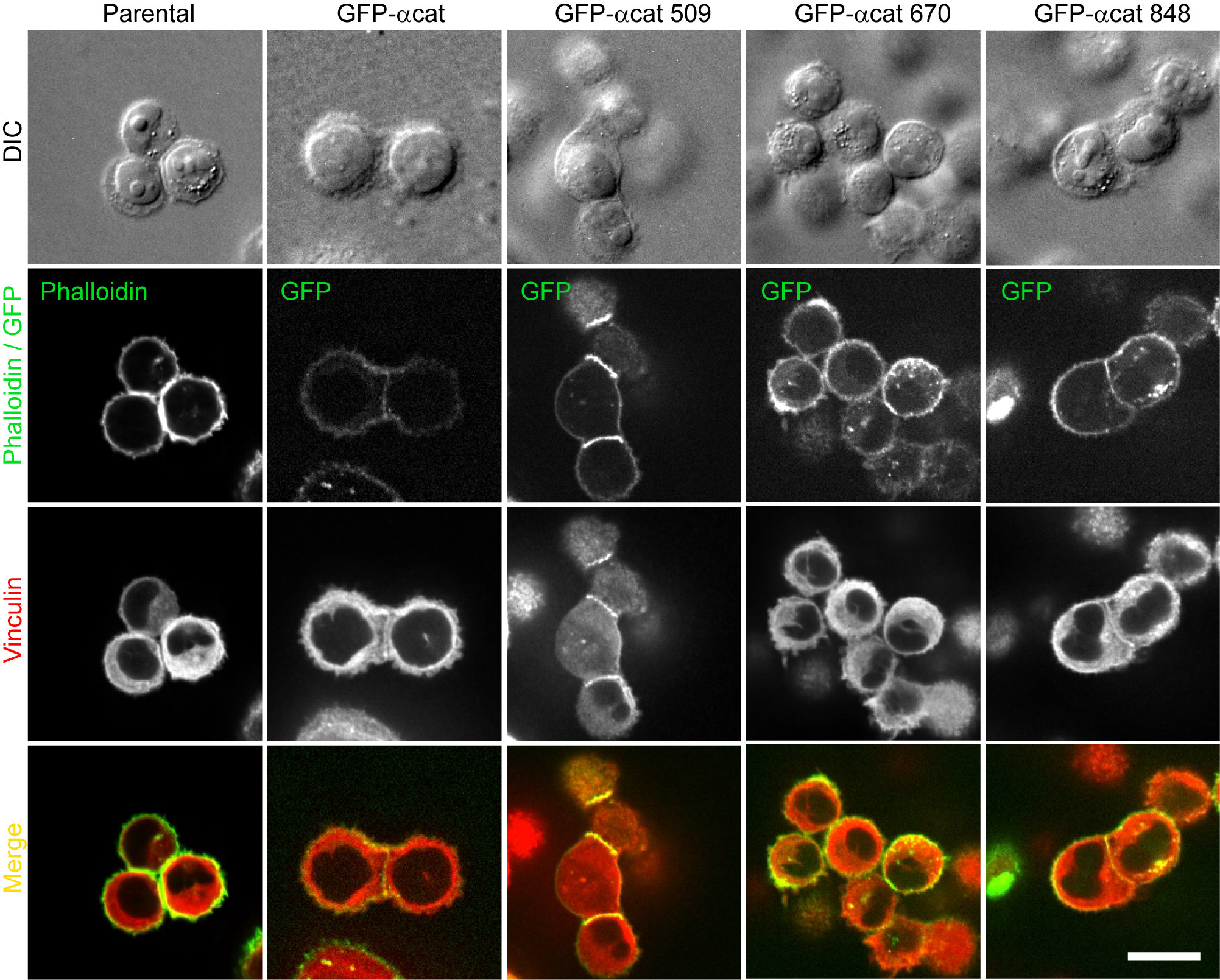

Supplement: Figure S3 — Immuno-fluorescence labeling of vinculin for cell aggregation analysis shown in Figure 6D . (TIF) [file pone.0055069.s003.tif]
